# Isolation and Genetic Characterization of Emerging H3N2 Canine Influenza Virus in Guangdong Province, Southern China, 2018–2021

**DOI:** 10.3389/fvets.2022.810855

**Published:** 2022-03-11

**Authors:** Jiajun Ou, Feiyan Zheng, Jiaojiao Cheng, Shaotang S. Ye, Cundong Ye, Kun Jia, Gang Lu, Shoujun Li

**Affiliations:** ^1^College of Veterinary Medicine, South China Agricultural University, Guangzhou, China; ^2^Guangdong Provincial Key Laboratory of Prevention and Control for Severe Clinical Animal Diseases, Guangzhou, China; ^3^Guangdong Technological Engineering Research Center for Pet, Guangzhou, China; ^4^College of Tropical Agriculture and Forestry, Guangdong Agriculture Industry Business Polytechnic, Guangzhou, China

**Keywords:** canine influenza virus, emerging, H3N2, China, genetic characterization

## Abstract

H3N2 canine influenza virus (CIV) emerged in dogs in China or Korea around 2005 and was first reported in 2008. In 2015, H3N2 CIV was detected in the United States and caused a huge outbreak. To date, H3N2 CIV is continuously circulating in dog populations in China, Korea, and the United States. For continuous monitoring of H3N2 CIV in China, we collected 180 dog nasal swab samples and 196 cat nasal swabs from veterinary hospitals in Guangdong Province between 2018 and 2021. Six emerging H3N2 CIV strains were isolated. Following full genome sequencing and phylogenetic analyses, we found that A/canine/Guangdong/1-3/2018 and A/canine/Guangdong/1-3/2021 diverged from the reported sequences of the Chinese H3N2 CIV strains. Moreover, we found that these H3N2 CIV strains belong to the group that contains US and northern China CIV strains in 2017 and 2019 and dominate in the dog population until 2021.

## Introduction

Influenza is a highly contagious and acute infection that usually occurs in the upper respiratory tract and has been detected in many vertebrate hosts worldwide. The recognized “mixing vessel” hosts for human influenza A virus (IAV) include pigs and birds, but recent data suggest that dogs and cats may also play such roles. As human companion animals, dogs and cats are exposed to the risk of influenza virus infection.

Although a few epidemiological and experimental infection studies in the 1970s indicated that dogs could be infected with IAV, the IAV strain was not isolated from dogs until 2004. This virus is now known as canine influenza virus (CIV), which is responsible for canine influenza (CI), a respiratory disease with the clinical features of cough, sneeze, fever, and nasal discharge in dogs (1, 2). CIV was classified into H3N8 and H3N2 subtypes, which were derived from interspecies transmission of equine and avian influenza virus, respectively. H3N8 CIV was first documented in the United States in 2004 and was later found in the United Kingdom and Australia (3–5). H3N2 CIV was first reported in Korea in 2007. Subsequently, H3N2 CIV was also reported in China by our laboratory, indicating that this virus was circulating in the Chinese dogs in 2006 (6, 7). In 2015, H3N2 CIV emerged in the United States, causing a huge outbreak. Sequencing results and further analysis indicated that the virus might be introduced from Korea. In China, H3N2 CIV was detected in different dog populations, including pet dogs, farmed dogs, stray dogs, and even in Tibetan mastiffs, with a broad geographical distribution from southern China to northern China (7, 8). The RNA polymerase of IAV lacks proofreading ability, and IAV mutates and evolves continually, which could also be observed in CIV.

As a potential “mixing vessel,” cats are sensitive to some IAVs isolated from humans, birds, and seals, which usually only cause subclinical infections or mild fever. In addition, the fact that avian influenza virus subtypes H5N1, H5N6, and H7N2 naturally infect tigers, leopards, and domestic cats and have infected related species through experimental inoculation shows that avian influenza virus has a strong pathogenicity to cats and the ability to cause systemic diseases (9–11). In addition, there have been reports of cross-species transmission of avian influenza viruses, IAV, CIV, and equine influenza virus to cats. However, since 2016, there have been no epidemiological surveys on cat infect influenza viruses in China (12, 13).

The cross-species spread of influenza viruses is an ongoing public health threat. Dogs and cats are vulnerable to a variety of influenza virus strains and lead to symptoms of varying degrees and viral shedding, and coinfection may lead to genetic recombination between virus strains. Dogs and cats, as companion animals, may become vectors for the spread of influenza virus among family members and may become a source of a new influenza pandemic, posing potential public health problems. Therefore, it is important to investigate and genetically characterize IAV in cat and dog infections. In this study, we collected 180 dog nasal swab samples and 196 cat nasal swab samples from sick dogs and cats in China from 2018 to 2021, detected 11 CIV-positive samples in dog nasal swab samples, and successfully isolated 6 strains of H3N2 CIV.

## Materials and Methods

### Samples Collection and Preparation

From 2018 to 2021, 180 dog nasal swab samples and 196 cat nasal swab samples from sick dogs and cats with respiratory disease were collected in veterinary hospitals in Guangdong Province in southern China. We collected two tubes for each nasal swab, and each tube contained 1 mL of normal saline. After collection, these samples were immediately stored at −80°C for further use.

### RNA and DNA Extraction, Virus Detection, and Screening

The pet hospital used a commercial reverse transcriptase–quantitative polymerase chain reaction (RT-qPCR) kit (Anheal, China) or an immunochromatographic strip kit (RapiGEN, South Korea) to detect the presence of IAV RNA/antigen in 24 dog nasal swab samples. We then used RT-PCR on the remaining samples to detect the presence of influenza virus RNA.

Total RNA was extracted from the nasal swab samples or virus stocks using TRIzol (Takara, Japan) according to the manufacturer's instructions. For detection of IAV, 7 μL of the extracted RNA was then subjected to cDNA synthesis using a HiScript II 1st Strand cDNA Synthesis Kit (Vazyme, Nanjing, China) with Uni12 primer (AGCAAAGCAGG) as the reverse transcription primer. For detection of canine parainfluenza virus (CPIV) and canine distemper virus (CDV), random primers were used as reverse transcription primers. Viral DNA was extracted from nasal swab samples using a MiniBEST Viral RNA/DNA Extraction Kit version 5.0 (Takara, Japan) according to the manufacturer's instructions for the detection of canine parvovirus (CPV) and canine adenovirus type 2 (CAV-2). IAV RNA was detected by PCR using universal primers targeting the M segment for cat nasal samples and targeting the hemagglutination assay (HA) segment for dog nasal samples. The DNA fragments with the expected size on 1% agarose gel electrophoresis were purified and then cloned into pCloneEZ-TA (Clone Smarter, USA) and sequenced (BGI, China). Detection primers are listed in Table 1.

**Table 1:** Primers used for virus detection and genome sequencing.

**Virus**	**Primer**	**Primer sequence (5^**′**^to 3^**′**^)**	**Gene**	**Target sequence size (bp)**
RT-PCR for detection				
Feline influenza virus	FIV-F	AGYCTTCTAACCGAGGWCGAA	M	226 bp
	FIV-R	TACGCTGCAGTCCTCGYTCA		
Canine influenza virus	CIV-F	CAAGCACTAATCAAGAACAAAC	HA	544 bp
	CIV-R	TCTGCTGCTTGTCCTGTACCTT		
Canine adenovirus type 2	CAV-2-F	CGCTGARCAYTACTACCTTGTCTATATTTATG	E3	1,020 bp
	CAV-2-R	GGTAGAGCWCTTCGTGTCCGCTT		
Canine parainfluenza virus	CPIV-F	ACAAAAATGTCATCCGTGCT	N	386 bp
	CPIV-R	ATCTCTCCACGGCTCATACC		
Canine distemper virus	CDV-F	GCTGGTTGGAGAATAAGG	N	586 bp
	CDV-R	CCAACTCCCATAGCATAA		
Canine parvovirus	CPV-F	CAGGTGATGAATTTGCTACA	Vp2	630 bp
	CPV-R	CATTTGGATAAACTGGTGGT		
**RT-PCR for genome sequencing**				
Canine influenza virus	CIV-PB2-F	AGCGAAAGCAGGTC	PB2	2,341 bp
	CIV-PB2-R	AGTAGAAACAAGGTCGTTT		
	CIV-PB1-F	AGCGAAAGCAGGCA	PB1	2,341 bp
	CIV-PB1-R	AGTAGAAACAAGG		
	CIV-PA-F	AGCGAAAGCAGGTAC	PA	2,233 bp
	CIV-PA-R	AGTAGAAACAAGGTACTT		
	CIV-HA-F	AGCAAAAGCAGGGG	HA	1,765 bp
	CIV-HA-R	AGTAGAAACAAGGGTGTTTT		
	CIV-NP-F	AGCGAAAGCAGGGTA	NP	1,565 bp
	CIV-NP-R	AGTAGAAACAAGGGTATTTTT		
	CIV-NA-F	AGCGAAAGCAGGAGT	NA	1,467 bp
	CIV-NA-R	AGTAGAAACAAGGAGTTTTTT		
	CIV-M-F	AGCGAAAGCAGGTAG	M	1,027 bp
	CIV-M-R	AGTAGAACAAGGTAGTTTTT		
	CIV-NS-F	AGCGAAAGCAGGGTG	M	890 bp
	CIV-NS-R	AGTAGAAACAAGGGTGTTTT		

*F, forward primer; R, reverse primer*.

### Virus Isolation and Whole Genome Sequencing

Positive nasal swab samples were processed for virus isolation in 9-day-old SPF embryonated chicken eggs for 48 h, and amniotic allantoic fluids were tested for IAV by HA using 1% chicken red blood cells. The positive samples were divided into 500-μL tubes and stored at −80°C.

The viral genome was obtained by PCR using universal primers targeting each segment of CIV. The primers used for genome sequencing are listed in Table 1. The DNA fragments with the expected size on 1% agarose gel electrophoresis were purified and then cloned into pCloneEZ-blunt (Clone Smarter, USA) and sequenced (BGI, China), and introduced into *Escherichia coli DH5*α-competent cells (Weidi, China) by transformation and sequenced.

### Sequences Analysis and Phylogenetic Analyses

After culturing for 12 h, bacterial clones were picked and identified by PCR; the positive *E. coli* clones were identified and sent for sequencing. Using the BLAST tool in the NCBI database, the sequence data were analyzed to confirm whether the sequenced *E. coli* clones contained the nucleotide sequence of CIV. The raw sequencing data were assembled and processed using SeqMan 7.1.0 and then aligned with other H3N2 CIV strains from China, the United States, and Korea using BioEdit 5.0.7.0. A comparison between their HA and NA proteins was performed. A comparison between their HA and NA proteins was performed. After estimation by “Find Best DNA Models,” phylogenetic trees were established with MEGA 6.0 using the maximum likelihood method with JTT+G model, based on the bootstrap values of 1,000 replicates.

## Results

### Virus Detection and Isolation

From 2018 to 2021, a total of 276 nasal swabs were collected from sick dogs and cats with respiratory disease in pet hospitals in Guangdong Province, southern China. We detected 11 nasal swab samples (6.11%) from positive dogs by RT-PCR, of which three were positive samples detected as CIV RNA/antigen-positive and were collected from different veterinary hospitals. To determine whether there was coinfection with other respiratory viruses, positive samples were detected for CDV, CPV, CAV-2, and CPIV by PCR. However, we did not detect the presence of other viruses in these nasal swabs. Moreover, we did not detect IAV RNA in 196 cat nasal swabs.

From the positive samples, six influenza viruses were isolated after the third passage using SPF eggs. Through sequencing, six influenza viruses corresponded to H3N2 subtype CIV (Table 2). The sequences of GD strains in this study were deposited in the GenBank database (A/canine/Guangdong/1/2018, A/canine/Guangdong/2/2018 A/canine/Guangdong/3/2018, A/canine/Guangdong/1/2021, A/canine/Guangdong/2/2021 and A/canine/Guangdong/3/2021: MK119981-MK120004 and A/OK639101-OK639112).

**Table 2:** Information on the samples collected from dogs in this study.

**Sample ID**	**Isolate**	**Isolation source**	**Collection date**	**Host**	**Detection method**	**Gender**	**Age**	**Clinical signs**
F1	A/canine/Guangdong/1/2018	Nasal swabs	2018/5/14	Corgi	RT-qPCR	Female	5 months	Fever, runny nose, cough, nasal congestion
W2	A/canine/Guangdong/2/2018	Nasal swabs	2018/5/16	Pomeranian	RT-qPCR	Male	10 months	Fever, runny nose, nasal congestion
J3	A/canine/Guangdong/3/2018	Nasal swabs	2018/5/17	Corgi	Immunochromatographic strip kit	Male	3 months	Fever, runny nose, cough, nasal congestion
A4	A/canine/Guangdong/1/2021	Nasal swabs	2021/5/2	Golden Retriever	RT-PCR	Female	6 years	Fever, runny nose, cough, nasal congestion
A5	A/canine/Guangdong/22021	Nasal swabs	2021/5	Chinese garden dog	RT-PCR	Female	3 years	Fever, runny nose, cough, nasal congestion
A6	A/canine/Guangdong/3/2021	Nasal swabs	2021/5/27	Pomeranian	RT-PCR	Male	5 months	Fever, runny nose, cough, nasal congestion

### Molecular Characteristics of the H3N2 CIV

To study the molecular characteristics of the H3N2 CIV genome, we amplified HA and NA segments of two CIV strains and eight-segment genome coding sequences (CDS) of four CIV strains. The nucleotide similarities of eight-segment sequence CDS of H3N2 CIV strains were calculated (Table 3). The HA segment nucleotide similarities of A/canine/Guangdong/1-3/2018 and A/canine/Guangdong/1-3/2021 ranged from 98.6% to 99.9%, and the HA segment nucleotide similarities of GD strains in this study with all H3N2 CIVin NCBI database ranged from 95.4% to 99.8%. Intriguingly, when we compared the HA sequences of A/canine/Guangdong/1-3/2021 with those of domestic strains in China, we found that the nucleotide similarities ranged from 95.4 to 99%, which were lower than those of strains from the United States and Korea. The NA segment of H3N2 CIV strains isolated in this study showed 94.99–99.9% nt identities with H3N2 CIV strains. However, the NA segment of A/canine/Guangdong/1-3/2021 had the greatest nucleotide similarities with A/canine/Texas/21-011409-001/2021 (H3N2) and other US CIV strains.

**Table 3:** The nucleotide similarities of complete genome sequences of H3N2 CIV strains.

**Segment**	**The nucleotide similarities of all H3N2 CIV**	**The nucleotide similarities of H3N2 CIV isolated from China**	**The nucleotide similarities of H3N2 CIV isolated from this study**
PB2*	96–99.9%	96–99.9%	99.2–99.9%
PB1*	96–99.9%	96–99.9%	99.4–99.7%
PA*	96.8–99.9%	96.8–99.9%	99.5–100%
HA	95.4–99.7%	95.4–99.7%	98.6–99.9%
NP*	96.3–99.7%	96.3–99.7%	99.3–99.9%
NA	94.9–99.9%	94.9–99.9%	99.4–99.7%
M*	96.2–99.9%	96.2–99.9%	99.3–99.8%
NS*	95.5–100%	96–100%	99.3–99.9%

*Aligned the eight-segment sequences of the six strains of this study.

In the analysis of the comparison of amino acid sequences of all H3N2 CIV strains in the NCBI database (Table 4), we found that the HA amino acid sequences of H3N2 CIV strains in this study all had V128I mutations, and GD strains in 2018 had N187S, A289S, N/T328S, and N481S mutations. GD strains in 2021 have T/N3I, G16S, V128I, A289S, and M/I552 L mutations in the HA protein. In the NA protein, A/canine/China/Guangdong/1/2018 and A/canine/Guangdong/2/2018 had two amino acid substitutions (V/I20A, N43K), A/canine/Guangdong/3/2018 had three amino acid substitutions (V/I20A, A/R30T, N43K), A/canine/Guangdong/1/2021 had one amino acid substitution (N/K330D), and A/canine/Guangdong/2/2021 had three amino acid substitutions (I17L, P121H, L256F). In addition, NP proteins in the GD 2018 and 2021 strains all had the unique substitution R293K. In addition, we found that the PB2 protein of GD strains in 2018 had 508Q and 559N amino acid substitutions, which only exist in US and Korean CIV strains.

**Table 4:** HA and NA amino acid differences between the H3N2 CIV strains analyzed in this study and previously analyzed H3N2 CIV strains.

**Isolate**	**HA**								**NA**						
	**3**	**16**	**128**	**187**	**289**	**328**	**481**	**552**	**17**	**20**	**30**	**43**	**121**	**256**	**330**
A/canine/Guangdong/1/2018	—	—	V → I	N → S	A → S	N/T → S	G → S		—	V/I → A	—	N → K	—		—
A/canine/Guangdong/2/2018	—	—	V → I	N → S	A → S	N/T → S	—		—	V/I → A	—	N → K	—		—
A/canine/Guangdong/3/2018	—	—	V → I	—	—	N/T → S	—		—	—	A/R → T	—	—		—
A/canine/Guangdong/1/2021	T/N → I	G → S	V → I	—	A → S		—	M/I → L	—				—		N/K → D
A/canine/Guangdong/2/2021	T/N → I	G → S	V → I	—	A → S		—		I → L				P → H	L → F	
A/canine/Guangdong/3/2021	T/N → I	G → S	V → I	—	A → S		—		—				—		

### Phylogenetic Analysis of the H3N2 CIV

**Figure 1:**
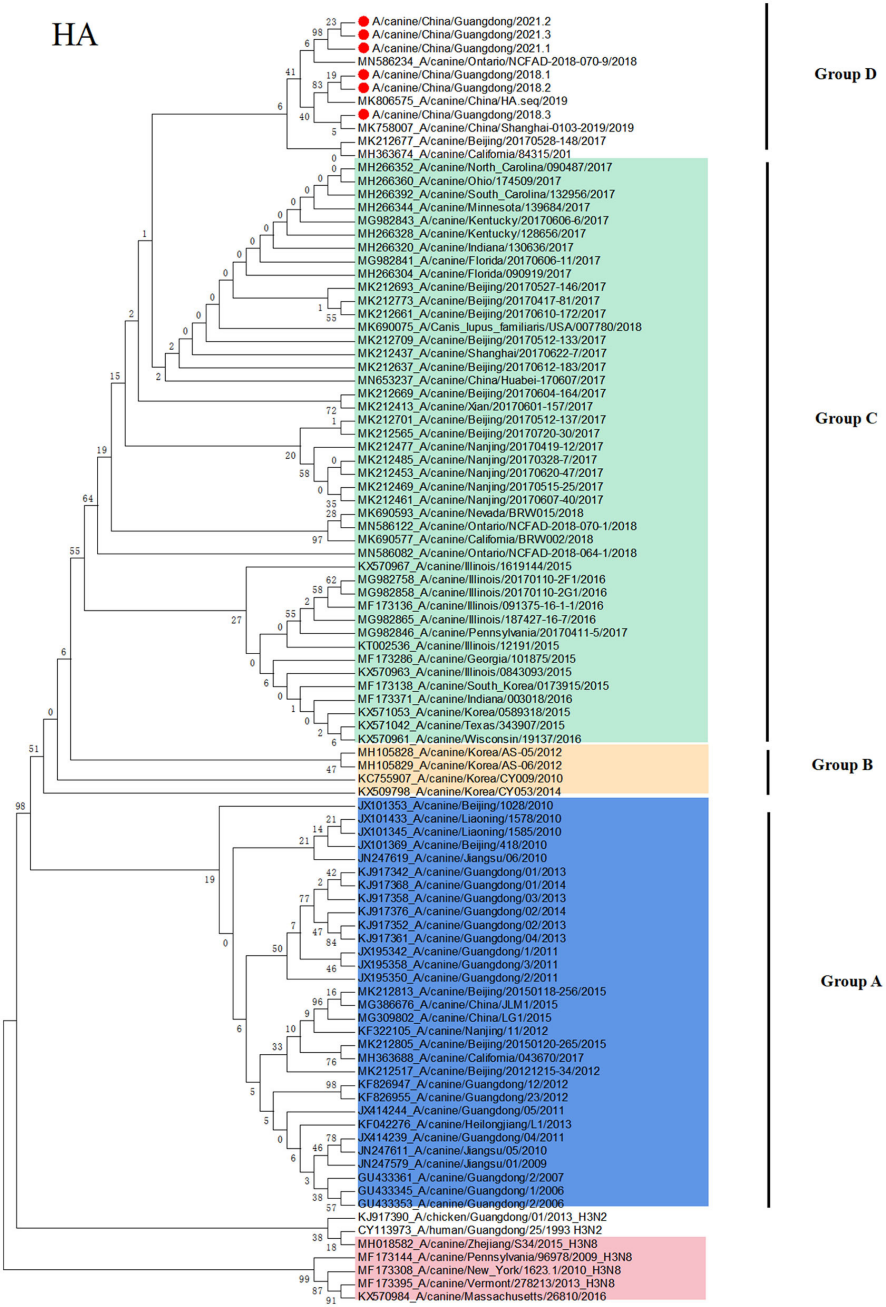
Phylogenetic analysis of H3N2 CIV based on the HA protein. Colors indicate H3N8 CIV (red) and isolates from different geographical regions of H3N2 CIV originating from the United States (green), Korea (yellow), and China (blue). The six Chinese strains sequenced in this study are indicated by red circles.

**Figure 2:**
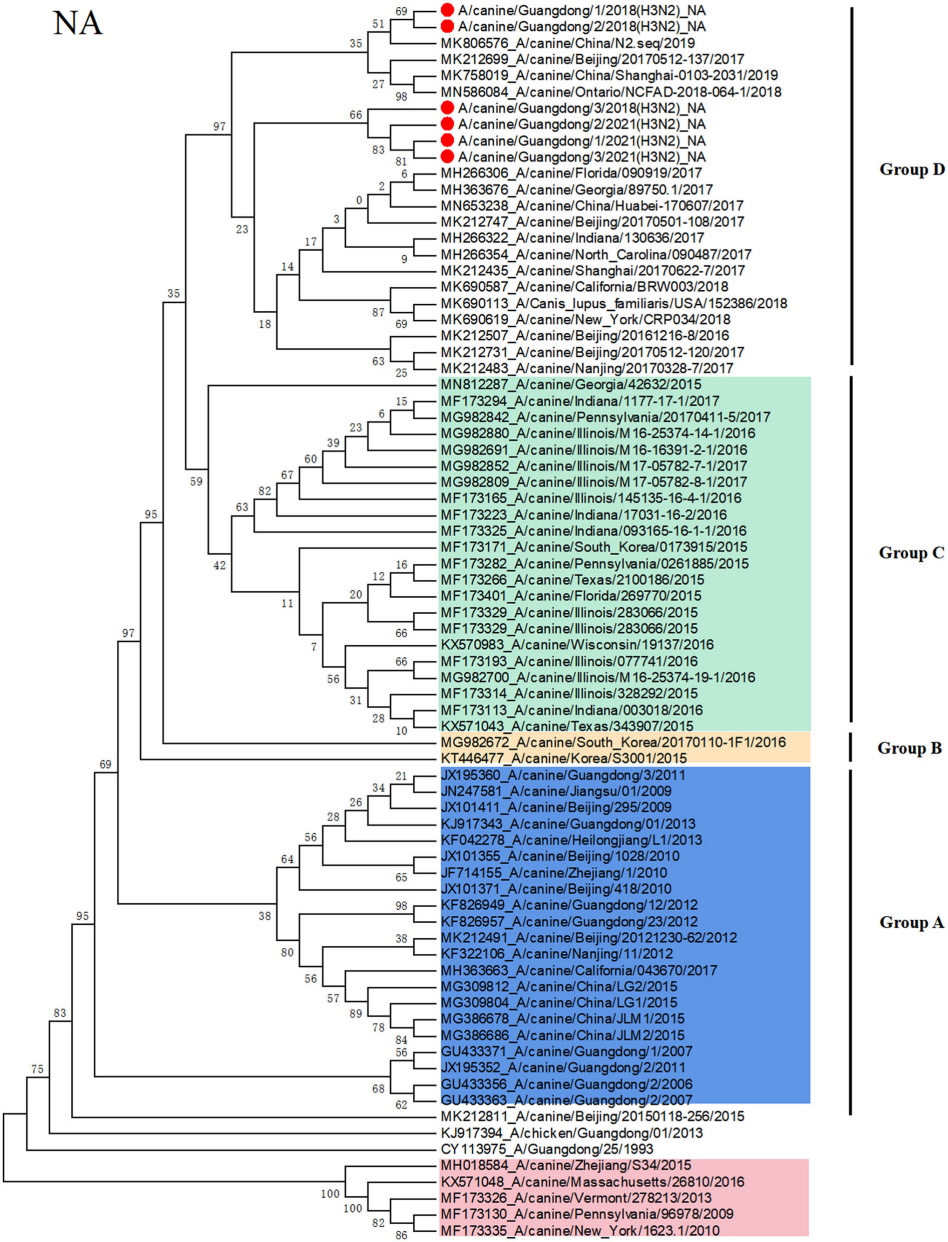
Phylogenetic analysis of H3N2 CIV based on the NA protein. Colors indicate H3N8 CIV (red) and isolates from different geographical regions of H3N2 CIV originating from the United States (green), Korea (yellow), and China (blue). The six Chinese strains sequenced in this study are indicated by red circles.

To clarify the origin of these viruses, we conducted phylogenetic analyses of the HA and NA genes (Figures 1, 2). Based on the phylogenetic analysis, we found that the HA and NA genes of H3N2 CIV can be divided into four groups: group A is Chinese CIV strains, group B is Korean CIV strains, group C is mainly American strains and northern China strains in 2017, and group D is mostly American strains and domestic strains isolated after 2018. Furthermore, the HA and NA proteins of A/canine/Guangdong/1-3/2018 and A/canine/China/Guangdong/1-3/2021 were divided into group D, which was closely related to the A/canine/Ontario/NCFAD-2018-070-16/2018 strain and Chinese strains after 2018. In addition, the A/canine/Guangdong/1-3/2021 strain formed a separate branch, which is far from the H3N2 CIV isolates reported in China before 2018. The NA gene of H3N2 CIV isolated in this study was also grouped into US H3N2 CIV isolates and far from the H3N2 CIV isolates reported in China.

## Conclusions

Since 2006, Chinese H3N2 CI strains have always belonged to unique branches, and there is no evidence of invasive US strains. However, in this study, we isolated emerging H3N2 CIV strains from sick dogs in China, and nucleotide identity analyses revealed that H3N2 CIV A/canine/Guangdong/1-3/2018 and A/canine/Guangdong/1-3/2021 are divergent from reported sequences in China before 2018. None of the six dogs and owners have been abroad and leaving Guangdong province in 2018, and the owners walk the dog at least once a day. However, we did not detect any influenza virus in cat nasal samples in Guangdong Province. In order to avoid the problem of primer specificity, we later also used CIV primers to detect 196 cat nasal swab samples, and the results were the same as before. Moreover, we have also verified that FIV primers can detect H3N2 FIV strains in our laboratory. As the three strains CIV of 2021 are from the same region and their HA and NA sequences are highly homologous, we amplified eight CDSs of one strain and only HA and NA of the other two strains. In addition, through phylogenetic analyses of the HA and NA genes, we found that the HA and NA genes of H3N2 CIV A/canine/Guangdong/1-3/2018 and A/canine/Guangdong/1-3/2021 were grouped in US isolates and far from Korean and Chinese isolates. Although the northern China strains in 2017 are classified into the same clade as the US isolates, A/canine/Guangdong/1-3/2018 and A/canine/Guangdong/1-3/2021 are classified into the same clade with US isolates A/canine/Ontario/NCFAD-2018-070-9/2018 and northern China isolate strains in 2017 and 2019. It is not ruled out that these strains may have evolved from northern China and are evolving in the same direction as the strains in the United States. Furthermore, these emerging H3N2 CIV strains in Guangdong had unique amino acid substitutions of HA and NA amino acid sequences compared with other Chinese isolates and US isolates. Although there was no mutation in the antigenic site, these strains were transmitted for some time, and some amino acid positions of the antigen protein changed.

In China, the prevention and control of CIV and FIV are relatively weak; most owners are not aware of the existence of the virus, and most people complain about the cost of pet hospitals. Moreover, there is still a lack of commercial vaccines, antibodies, and targeted drugs for CIV and FIV. Pet hospitals can only provide symptomatic treatment and broad-spectrum antiviral treatment for dogs and cats instead of specific treatment options. This has undoubtedly increased the difficulty of our treatment, investigation, prevention, and control of CIV and FIV.

In conclusion, in this study, we isolated six emerging H3N2 CIV strains and sequenced their genome sequences of the H3N2 CIV strains, A/canine/Guangdong/1-3/2018 and A/canine/Guangdong/1-3/2021, from China, which are divergent from the reported sequences of Chinese H3N2 CIV strains and grouped into US isolates. Although there was an outbreak of CIV infection in northern China in 2017, we did not detect CIV in dogs during the 2017 CIV outbreak. In addition, from the phylogenetic tree of HA protein, the GD strains and the US strains were separated into a single strain, and the Chinese strain in 2017 was farther in genetic distance. This clade could have originated from H3N2 CIVs circulating in the US and southern China strain. Moreover, no China strains belonging to group D known before 2016 were detected and dominant in the dog population until 2021. Most Asian countries such as Japan and Thailand have continuous detection of CIV, but they presented many gaps of sequence information as they just detected only the genome sequences and did not characterize CIV strains in their country (14, 15). It is suggested that we should facilitate the informative data of the CIV diversity and requested intensive monitoring and strengthen the monitoring of disease in dogs during cross-border transport and continue to monitor the CIV in China for a long period of time to observe the recombination of domestic and foreign strains. It is also important to monitor whether the coexistence of the two origin viruses has a greater impact on dogs; this requires further research.

## Data Availability Statement

The original contributions presented in the study are included in the article/supplementary materials, further inquiries can be directed to the corresponding author/s.

## Author Contributions

JO performed the experiments and analyzed the data. CY, KJ, GL, and SL participated in whole study design, coordinated the study, and prepared the manuscript and manuscript revision. FZ, SY, and CY assisted with manuscript revision. JC assisted with conducting the experiments. All authors have read and agreed to the published version of the manuscript.

## Funding

This work was supported by the Natural Science Foundation of Guangdong Province (2018B030311037); the Special Fund for Agro-Scientific Research in the Public Interest (201303042); the National Key Research and Development Program of China (2016YFD0501004); Guangdong Provincial Key Laboratory of Prevention and Control for Severe Clinical Animal Diseases (No. 2017B030314142); and Guangdong Provincial Key Scientific Research Platform and Young Innovative Talents Scientific Research Project (2018GKQNCX148).

## Conflict of Interest

The authors declare that the research was conducted in the absence of any commercial or financial relationships that could be construed as a potential conflict of interest.

## Publisher's Note

All claims expressed in this article are solely those of the authors and do not necessarily represent those of their affiliated organizations, or those of the publisher, the editors and the reviewers. Any product that may be evaluated in this article, or claim that may be made by its manufacturer, is not guaranteed or endorsed by the publisher.
